# Broad-Spectrum Photo-Antimicrobial Polymers Based on Cationic Polystyrene and Rose Bengal

**DOI:** 10.3389/fmed.2021.641646

**Published:** 2021-05-24

**Authors:** Raquel Gavara, Rosa de Llanos, Vanesa Pérez-Laguna, Carla Arnau del Valle, Juan F. Miravet, Antonio Rezusta, Francisco Galindo

**Affiliations:** ^1^Departamento de Química Inorgánica y Orgánica, Universitat Jaume I, Castellón, Spain; ^2^Unidad Predepartamental de Medicina, Universitat Jaume I, Castellón, Spain; ^3^Instituto de Investigación Sanitaria Aragón, Departamento de Microbiología, Hospital Universitario Miguel Servet, Zaragoza, Spain; ^4^Universidad de Zaragoza, Zaragoza, Spain

**Keywords:** ESKAPE, antimicrobials, polystyrene, broad-spectrum, photodynamic inactivation, singlet oxygen

## Abstract

New strategies to fight bacteria and fungi are necessary in view of the problem of iatrogenic and nosocomial infections combined with the growing threat of increased antimicrobial resistance. Recently, our group has prepared and described two new readily available materials based on the combination of Rose Bengal (singlet oxygen photosensitizer) and commercially available cationic polystyrene (macroporous resin Amberlite® IRA 900 or gel-type resin IRA 400). These materials showed high efficacy in the antimicrobial photodynamic inactivation (aPDI) of *Pseudomonas aeruginosa*. Here, we present the photobactericidal effect of these polymers against an extended group of pathogens like *Escherichia coli, Enterococcus faecalis, Staphylococcus aureus*, and the opportunistic yeast *Candida albicans* using green light. The most interesting finding is that the studied materials are able to reduce the population of both Gram-positive and Gram-negative bacteria with good activity, although, for *C. albicans*, in a moderate manner. In view of the results achieved and especially considering the inexpensiveness of these two types of photoactive polymers, we believe that they could be used as the starting point for the development of coatings for self-disinfecting surfaces.

## Introduction

Nosocomial infections are growing in importance day by day and constitute a serious problem for public health, causing important human and economical loses. In the future, it is expected that bacterial and fungal infections will be a major cause of death worldwide (1). These infections are mainly originated by a growing number of bacteria and fungi with strong resistance to chemotherapeutical drugs, and special attention is paid to the development of strategies that deal with the well-defined group of ESKAPE pathogens (*Enterococcus faecium, Staphylococcus aureus, Klebsiella pneumoniae, Acinetobacter baumannii, Pseudomonas aeruginosa*, and *Enterobacter* species) (2).

Other important sources of nosocomial infections are opportunistic fungal pathogens, especially in immunocompromised patients (3). In particular, several *Candida* spp. are widely recognized as majorly responsible for the morbidity and mortality caused by opportunistic microbes in healthcare settings (4). Similarly, the emergence of *Candida* spp. resistant to antifungal drugs is also widely recognized and therefore has become a global health problem (5). Despite the intensive work carried out in order to develop alternatives to the current drug treatments (6, 7), the most realistic approach to fighting antimicrobial-resistant microorganisms continues to be the prevention of contagion.

Nosocomial infections arise mainly from the growth of microorganisms in surfaces in close contact to patients, for instance orthopedic implants, catheters, and gastroesophageal tubes. Therefore, the development of antimicrobial coatings engineered for use in medical devices is of great practical interest. Several strategies have been developed in the past to make surfaces with antimicrobial properties, and the literature is abundant in reviews about this topic (8, 9). Thus, it is possible to design surfaces with antifouling properties that inhibit the adherence of microorganisms by controlling, for example, the surface hydrophobicity (10). Also, there is relevant research on the development of coatings with intrinsic antimicrobial features by the incorporation of biocide compounds (11–13).

An emerging strategy to fight hospital-acquired infections is the so-called antimicrobial photodynamic inactivation (aPDI) (14–17). This approach has been developed in parallel with the photodynamic therapy (PDT) of cancer (18, 19), although in recent times it has attracted a renewed interest (14, 15, 20–26). It is based on the killing of microorganisms by reactive oxygen species (ROS), for instance singlet oxygen and radicals, which in turn are generated due to the absorption of light by a photosensitizer in the presence of oxygen. Since the mechanistic aspects of the processes involved are very well-described elsewhere, the reader is referred to any of the excellent reviews published in the literature about photosensitization (27–33).

Based on this strategy, we reported recently (34) on simple and inexpensive photosensitizing materials based on the ionic attachment of the anionic singlet oxygen photosensitizer Rose Bengal (RB) on commercial cationic polystyrene (Amberlite® IRA 900 and IRA 400). The materials previously described by our group were able to eradicate completely the population of *P. aeruginosa* under irradiation [reduction of 8 log_10_ colony forming units (CFU) per milliliter]. In the present work, we extend the evaluation of these materials as aPDI agents against other relevant pathogens as well as the yeast *C. albicans*. The results presented here indicate that these photoactive polymers could be good starting points for the development of coatings for medical devices that prevent hospital-acquired infections. It has to be noted that the use of ionic exchange for the preparation of photoactive materials can be traced back to the pioneering work of Williams et al. on polymers for photocatalytic applications (35).

The present investigation can be enclosed within the interdisciplinary emerging field of materials for aPDI, which use typically biopolymers or synthetic organic macromolecules as supports (20, 22, 36–39).

## Materials and Methods

### Synthesis and Characterization of the Polymeric Photosensitizers

The photosensitizing polymers RB@P_mp_ and RB@P_gel_ were prepared from RB sodium salt (Sigma-Aldrich) and the ion exchange resins Amberlite® IRA-900 (P_mp_) and Amberlite® IRA-400 (P_gel_), respectively (chloride forms, both from Sigma-Aldrich). The synthesis and characterization are reported elsewhere (34).

### Microorganisms and Growth Conditions

The Gram-positive bacterial strains *E. faecalis* ATCC 29212 and *S. aureus* ATCC 29213, Gram-negative *E. coli* ATCC 25922, as well as the yeast strains of *C. albicans* ATCC 10231 were acquired from the American Type Culture Collection (ATCC, Rockville, MD, USA).

Microorganisms seeded on Columbia Blood Agar (Oxoid® Madrid, Spain) were cultured aerobically overnight at 35°C.

### Antimicrobial Photodynamic Inactivation Experiments

The inoculum was prepared by adding colonies in distilled water (Gibco®, Thermo Fisher, Spain) and adjusted to 0.50 ± 0.03 on the McFarland scale for bacteria and to 5.00 ± 0.03 on the McFarland scale for *C. albicans* (microbial suspensions containing >10^8^ bacteria/ml and >10^6^ yeasts/ml, respectively).

Ten experimental groups for each strain were prepared with the inocula. They were prepared using 10 different RODAC plates and dropping a volume of 5 ml of the microbial suspensions into each one and then 200 mg of the photoactive polymer RB@P_mp_ (group I), or the same amount of control P_mp_ resin (without RB; group II), or 200 mg of the photoactive polymer RB@P_gel_ (group III), or the same amount of control P_gel_ resin (without RB; group IV), or no resin was added (group V). These five groups were subjected to irradiation, and in parallel, another five groups were kept in darkness as controls (groups VI to X).

Supplementary Figures 1A,C show the setup used.

The samples were shaken (mode: orbital 15 rpm; Grant Bio™ PS-M3D 3D Multi-Function Rotator) during the irradiation (groups I to V) or during the time corresponding to the irradiation period (groups VI to X).

The source light used was a light-emitting diode lamp (Showtec LED Par 64 Short 18 × RGB 3-in-1 LED, Highlite International B.V., Spain) emitting at 515 ± 10 nm (green range matching the excitation spectrum of RB in the polymers; Supplementary Figure 1D). Supplementary Figure 1B shows the LED emission spectrum. The irradiation was performed using a total light dose up to 200 J/cm^2^, keeping a 17-cm distance between the LEDs and the RODAC plates (light irradiance, 5.8 mW/cm^2^).

Final loading of RB in the polymers was 1.5 mg RB/g resin, that is, a concentration of 60 μg/ml or 5.9 × 10^−5^ M (200 mg of RB@P_mp_ or RB@P_gel_ in 5 ml of microbial suspension).

No incubation time after the addition of the polymers to the microbial suspension was used, that is, when the polymers are added is when *t* = 0 is established and the irradiation or darkness time begins to be counted.

Aliquots from the RODAC plates were taken every time equivalent to a 20-J/cm^2^ light dose (57.6 min of illumination or darkness) up to a maximum of 200 J/cm^2^ (9.6 h of illumination); the appropriate dilutions were made and they were seeded in blood agar plates and incubated overnight at 35°C. The antimicrobial effect was determined by counting the number of CFU per milliliter on the plate using the Flash & Go automatic colony counter (IUL, S.A, Spain). The aliquots had a volume of 10 μl (0.2% of the initial sample volume). The dilutions or the direct seeding in the plates for counting were carried out according to previous experiments in order to count the range {>0, <200} CFU/agar plate. Higher volumes of aliquots were taken in cases where, according to the preliminary experiments, the CFU number in the plates from the aliquot of 10 μl planted undiluted was 0 CFU/agar plate (i.e., bacterial or fungal growth is expected to be <100 CFU/ml; this equates to bacterial samples where the logarithmic reduction reaches or exceeds 6 log_10_ or ≥4 log_10_ for *C. albicans*).

In these cases, the volume removed was 100 μl (2% of the initial sample volume) and the maximum volume taken was 1 ml (20% of the initial sample volume) in the points where there were <10 CFU/ml (the logarithmic reduction reaches or exceeds 7 log_10_ in the bacterial samples or ≥5 log_10_ for the yeast samples).

All experiments were performed three times: five groups for irradiation + five groups for darkness (=10) for each type of polymer (×2); it was performed for each microorganism (×6) in three replicates of the experiment (×3). Graphs of the results and statistical analysis were done using GraphPad Prism 8. The results are expressed as mean and standard deviation. Differences between groups were compared by analysis of variance.

## Results and Discussion

The polymeric supports used in this study, Amberlite® IRA-900 and IRA-400, are commercially available ion exchange resins used in diverse fields, from catalysis to chromatography. They consist of cross-linked polystyrene with appended trimethylammonium groups (with chloride anions). The difference between both resins is the degree of cross-linking: Amberlite® IRA-900 (P_mp_) presents a high degree of cross-linking, and hence permanent porosity, giving rise to a macroporous structure. On the other hand, Amberlite® IRA-400 (P_gel_) presents a lower degree of cross-linking and lacks permanent porosity, thus presenting a gel-type structure in the presence of the appropriate compatible solvent. Preparation of the photo-antimicrobial conjugates involving these resins and RB was easily done by the exchange of chloride ions present in the original Amberlite® polymers (P_mp_ and P_gel_), by RB anions, yielding the final polymers RB@P_mp_ and RB@P_gel_, respectively. More details about the synthesis and characterization of the materials can be found in our previous work (34).

The photodynamic activity of the materials using green light (515 nm) was tested against two strains of Gram-positive bacteria such as *E. faecalis* and *S. aureus* and two strains of Gram-negative bacteria, specifically *E. coli* and *P. aeruginosa*. We have recently reported on the photodynamic activity of both Amberlite® polymers (P_mp_ and P_gel_) against *P. aeruginosa* (34), and therefore the results for this Gram-negative bacteria are included in the present work for comparison purposes. In addition, the photoactivity against *C. albicans* is also presented in this study in order to have a fungal representative. Overall, we present a broad-spectrum photo-antimicrobial analysis of these polymers based on cationic polystyrene and RB.

### Activity Against Gram-Positive Bacteria

**Figure 1:**
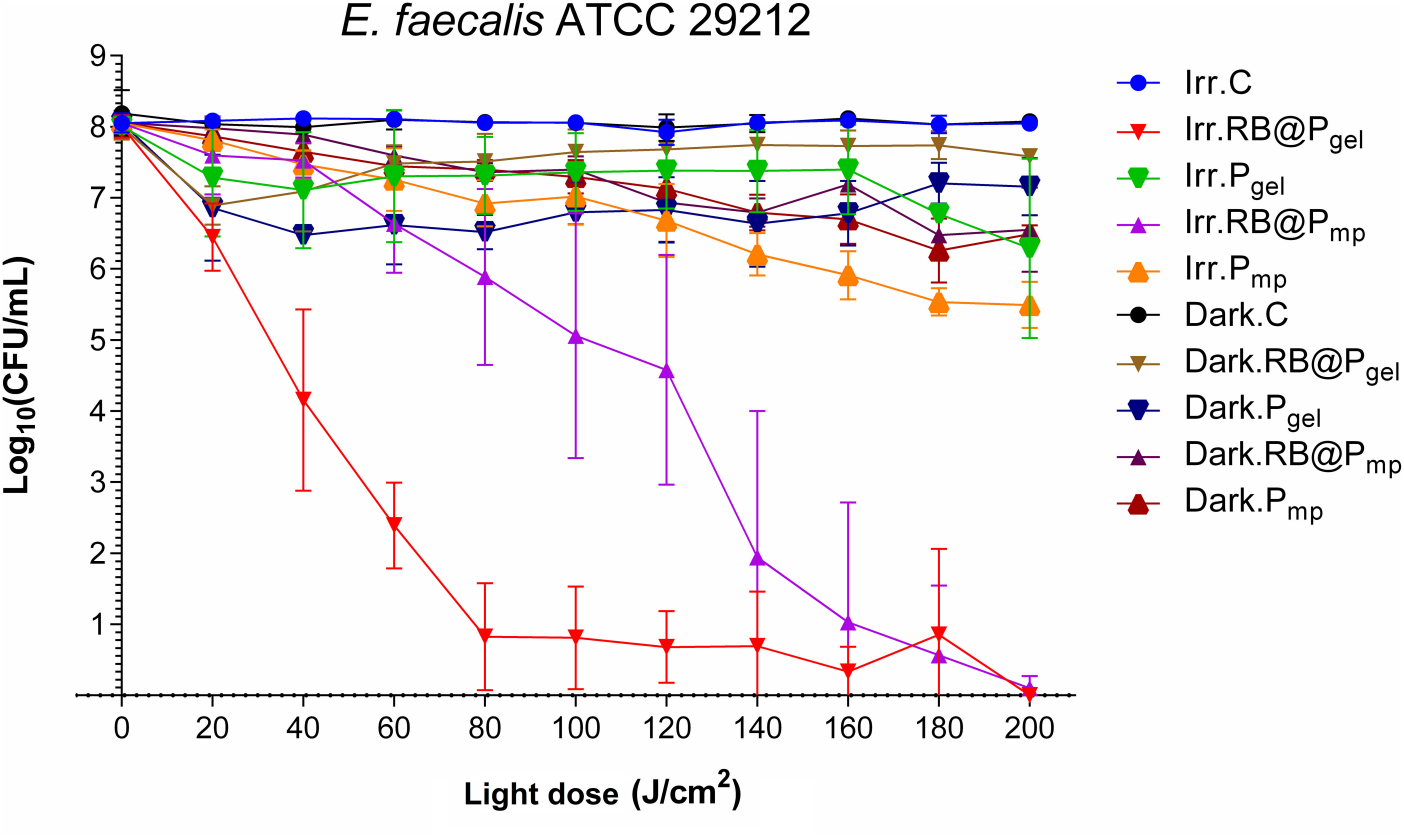
Survival curves corresponding to the photodynamic inactivation of *Enterococcus faecalis*. Every *point* is the average of three independent experiments. *Error bars* correspond to the standard deviations. Legend titles: *Irr*, irradiated samples; *Dark*, controls in the darkness; *C*, control, only microbial suspension; *RB@P*_*gel*_, Amberlite® IRA-400 (P_gel_) loaded with Rose Bengal (RB); *P*_*gel*_, P_gel_ resin without RB; *RB@P*_*mp*_, Amberlite® IRA-900 (P_mp_) loaded with RB; *P*_*mp*_, P_mp_ resin without RB.

Both RB@P_mp_ and RB@P_gel_ materials present a high efficiency against Gram-positive *E. faecalis* at a total light dose of 200 J/cm^2^, with a total eradication of the bacterium population (8 log_10_ CFU/ml). At lower exposures to light (100 J/cm^2^), differences between both polymers can be noticed, showing the gel matrix to have a better performance than the macroporous one (Figure 1 and Supplementary Figures 2, 3). Additionally, the corresponding controls in the dark as well as the polymeric matrices P_mp_ and P_gel_ without a photosensitizer show some activity, with log_10_ reductions of CFU per milliliter in the range of 0.5–2 units. This partial activity can be, in principle, attributed to the presence of ammonium groups in the polymeric matrices, which are known to have antimicrobial effects by triggering bacterial envelope destruction (40).

Several studies have described the photodynamic killing of planktonic suspensions of *E. faecalis* by different photosensitizing materials (Table 1 shows some representative examples). Although the different experimental setups used make a direct comparison of bibliographic data difficult, we would like to illustrate the effectiveness of our systems against different bacterial pathogens in the context of other materials studied for the same goal. It is worth noting the activity of chitosan nanoparticles functionalized with RB (CS-RB) (43) causing a notable reduction of *E. faecalis* viability. Moreover, the dark toxicity of the reported nanoparticles was significant, indicating that the cationic matrix is also playing an important role in such bactericidal effect.

**Table 1:** Representative examples reported in the literature of *Enterococcus faecalis* inactivation caused by photosensitizing materials.

**Photosensitizer**	**Support**	**Initial load (log_**10**_ CFU/ml)**	**Load reduction (Δlog_**10**_ CFU/ml)**	**References**
Indocyanine green	Nano-graphene oxide	5	2.81	(41)
Porphyrin	Magnetic silica NPs	5	5	(42)
Rose Bengal	Chitosan NPs	8	8	(43)
Rose Bengal	P_mp_ (IRA900)	8	8	This work
Rose Bengal	P_gel_ (IRA400)	8	8	This work

**Figure 2:**
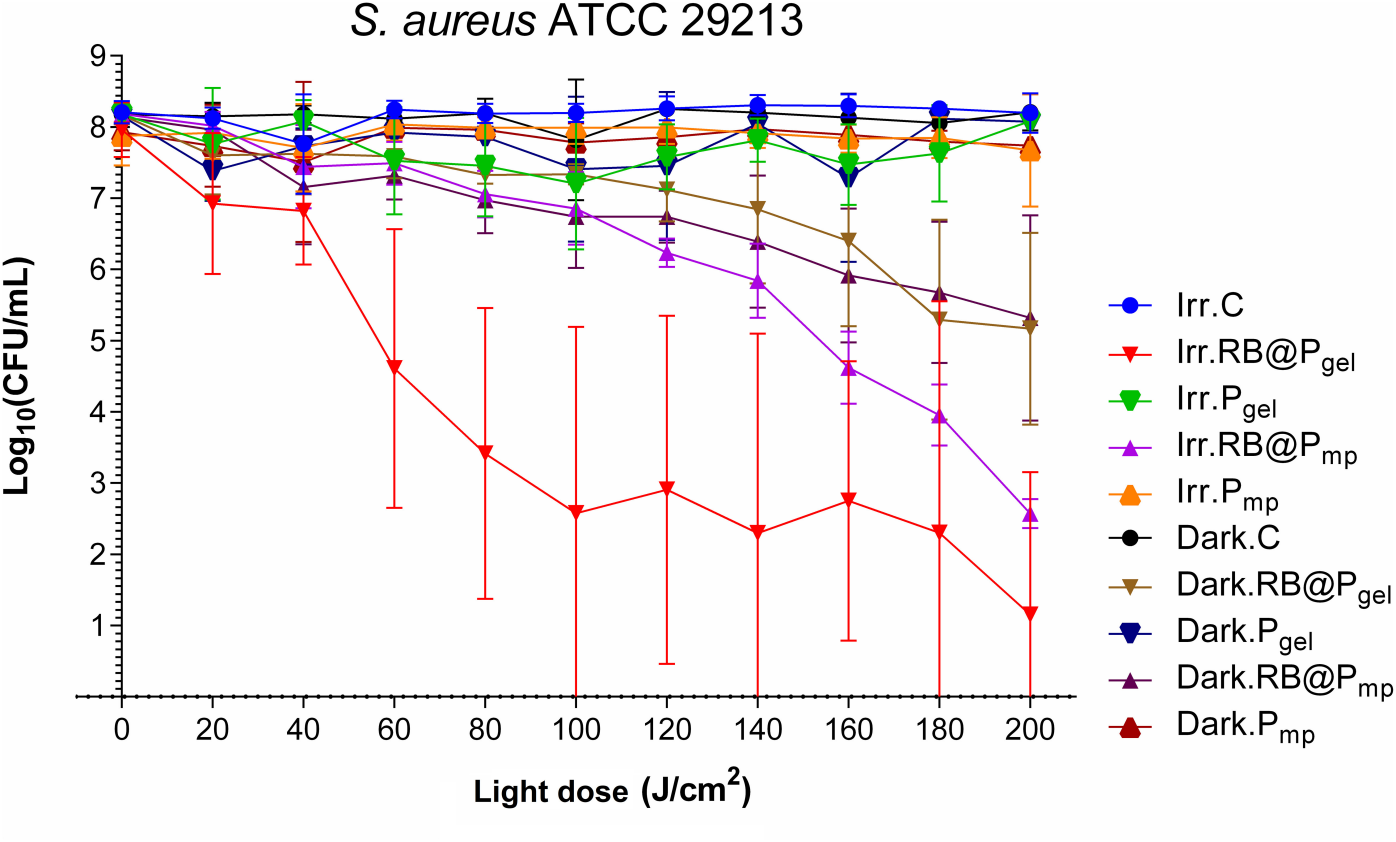
Survival curves corresponding to the photodynamic inactivation of *Staphylococcus aureus*. Every *point* is the average of three independent experiments. *Error bars* correspond to the standard deviations. Legend titles: *Irr*, irradiated samples; *Dark*, controls in the darkness; *C*, control, only microbial suspension; *RB@P*_*gel*_, Amberlite® IRA-400 (P_gel_) loaded with Rose Bengal (RB); *P*_*gel*_, P_gel_ resin without RB; *RB@P*_*mp*_, Amberlite® IRA-900 (P_mp_) loaded with RB; *P*_*mp*_, P_mp_ resin without RB.

For *S. aureus*, the bacterial viability reduction after irradiation is dependent on the polymer used. The activity for RB@P_gel_ is better than for RB@P_mp_ at lower light doses, but similar at 200 J/cm^2^ (5.5–7 log_10_) (Figure 2 and Supplementary Figures 2, 3). The results of the RB-containing polymers in the dark also demonstrate a significant activity, as denoted by a reduction of 3 log_10_ in the count of *S. aureus* population (at the end of the kinetics). It can be hypothesized that part of the dark toxicity of RB@P_gel_ and RB@P_mp_ could be originated by the fact that RB was recently found to be a potent inhibitor for SecA ATPase activity, which is essential in protein translocation in bacteria (44). Thus, if some photosensitizer is transferred from the polymers to the bacteria during the course of the experiments, this could originate some reduction of the CFU per milliliter. However, more experiments are needed in order to confirm this activity. This process seems very unlikely since, according to preliminary assays, no leaching out of RB takes place, as determined spectrophotometrically, after keeping both RB@P_mp_ and RB@P_gel_ submerged in water for several weeks.

The photoinactivation of this pathogen by different photoactive materials has been extensively reported in the literature. Some recent representative examples of planktonic studies are shown in Table 2. Typical reductions of the bacterial population range from 4 to 6 log_10_ CFU/ml. We have previously reported the notable activity of the hexanuclear molybdenum cluster [Mo_6_I_8_Ac_6_]^2−^ when loaded in the same polymeric matrices used in the present work for both Gram-positive and Gram-negative bacteria. These polymers exhibited a slightly better performance than RB@P_mp_ and RB@P_gel_, with a 7–8 log_10_ reduction in the populations of *S. aureus* (49). Some questions are still open regarding the use of molybdenum hybrid polymers for the coating of medical devices, in front of the RB-loaded polymers presented here, like the unknown toxicity of the molybdenum clusters as well as the higher cost of preparation.

**Table 2:** Recent examples reported in the literature of *Staphylococcus aureus* inactivation caused by photosensitizing materials.

**Photosensitizer**	**Support**	**Initial load (log_**10**_ CFU/ml)**	**Load reduction (Δlog_**10**_ CFU/ml)**	**References**
Porphyrin	Dipyrromethane polymeric films	7–7.8	4–5	(45)
Electropolymerizable Zn(II) porphyrin containing carbazoyl groups	Polymeric films from polymerization of the porphyrin	6	6	(46)
Methylene blue	Methacrylate polymer doped with montmorillonite	8–8.7	4.8	(47)
Rose Bengal	Sol–gel hybrid coatings based on alkyl silanes	4.4	4.4	(48)
${\left[Mo_{6}I_{8}Ac_{6}\right]}^{2-}$	P_mp_ (IRA900)	8	8	(49)
${\left[Mo_{6}I_{8}Ac_{6}\right]}^{2-}$	P_gel_ (IRA400)	8	7	(49)
Rose Bengal	P_mp_ (IRA900)	8	5.5	This work
Rose Bengal	P_gel_ (IRA400)	8	7	This work

### Activity Against Gram-Negative Bacteria

**Figure 3:**
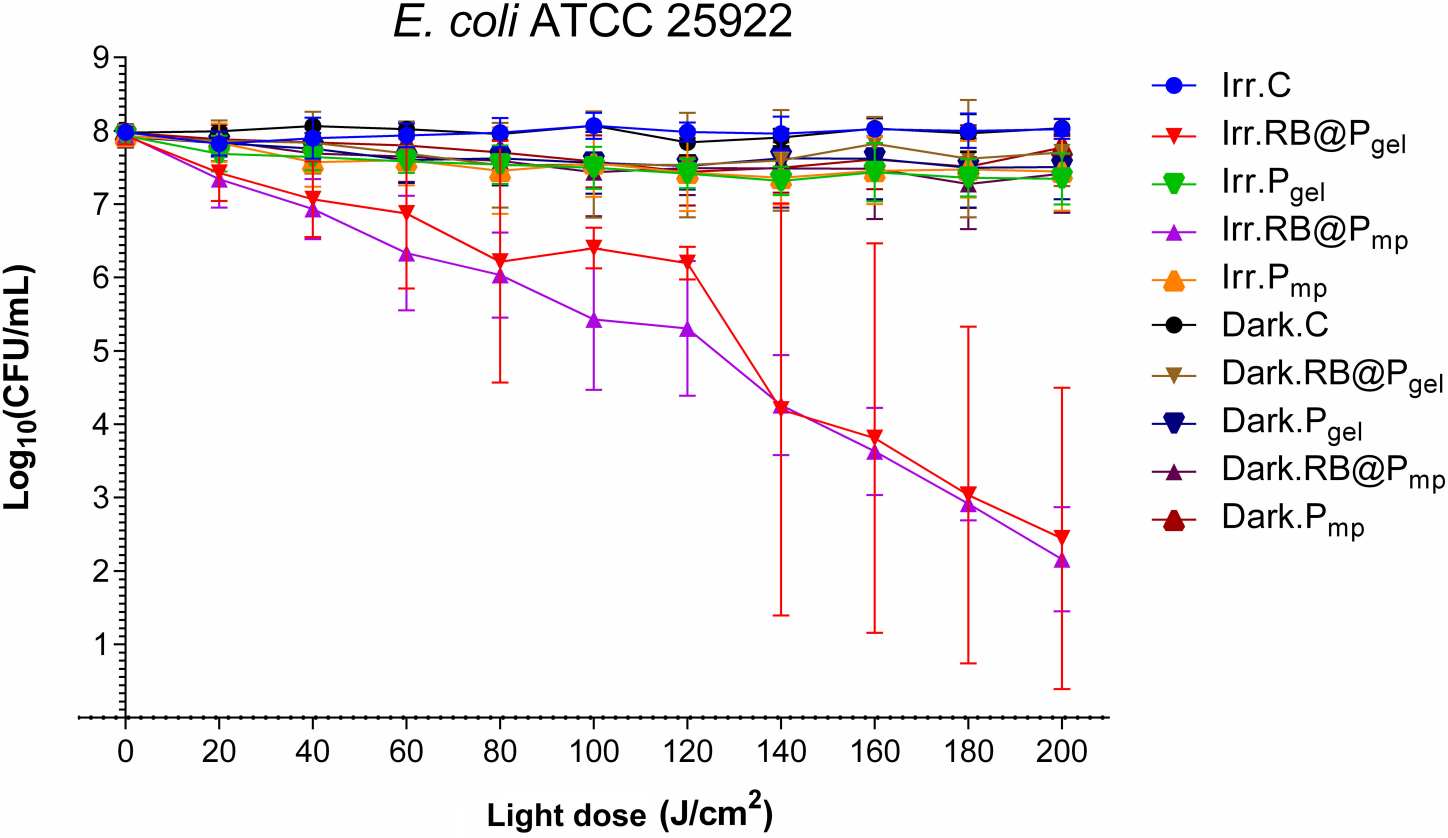
Survival curves corresponding to the photodynamic inactivation of *Escherichia coli*. Every *point* is the average of three independent experiments. *Error bars* correspond to the standard deviations. Legend titles: *Irr*, irradiated samples; *Dark*, controls in the darkness; *C*, control, only microbial suspension; *RB@P*_*gel*_, Amberlite® IRA-400 (P_gel_) loaded with Rose Bengal (RB); *P*_*gel*_, P_gel_ resin without RB; *RB@P*_*mp*_, Amberlite® IRA-900 (P_mp_) loaded with RB; *P*_*mp*_, P_mp_ resin without RB.

It is known that Gram-negative bacteria are more resistant to photodynamic inactivation than Gram-positive bacteria due to their highly organized outer wall (22). It has been reported that an effective inactivation of Gram-negative bacteria requires the presence of cationic photosensitizers, and in consequence, it has been found that RB is relatively inefficient against these bacteria in its free form, but highly effective in combination with adjuvants like cationic peptides (50) or core–shell silver–silica nanoparticles (51). It must be noted that the positive effect of cationic residues (not belonging strictly to the photosensitizer) was described earlier for chlorin e6 conjugated to poly-l-lysine (52). Thus, we decided to investigate the RB inhibitory effect when it is supported on the cationic Amberlite resins. The results obtained using RB@P_mp_ and RB@P_gel_ demonstrate that RB becomes an efficient photosensitizer against the Gram-negative bacteria *E. coli* at a total light dose of 200 J/cm^2^, with a reduction of CFU per milliliter of ~5.5 log_10_ units (Figure 3 and Supplementary Figure 3). In this case, no important differences were detected between the gel and macroporous polymers, as can be seen from the data at 100 J/cm^2^ (Supplementary Figure 2).

The photoinactivation of *E. coli* as a model of Gram-negative bacterium has been thoroughly studied in the past. Some recent examples using photosensitizing materials are shown in Table 3. Interestingly, Bilici et al. (53) reported a remarkable activity of indocyanine green loaded in superparamagnetic iron oxide nanoparticles. However, they combined photodynamic therapy with photothermal therapy to trigger antibacterial phototoxicity, which cannot be comparable with our system or any of the other studies presented in Table 3.

**Table 3:** Recent examples reported in literature of *Escherichia coli* inactivation caused by photosensitizing materials.

**Photosensitizer**	**Support**	**Initial load (log_**10**_ CFU/ml)**	**Load reduction (Δlog_**10**_ CFU/ml)**	**References**
Indocyanine green	Superparamagnetic iron oxide NPs	12	12	(53)
Porphyrin	Metal organic framework/cotton fabrics	8	6	(54)
Porphyrin	Silica-coated magnetite NPs	6	3.1	(55)
Cationic Pd(II) porphyrin	Polyacrylamide hydrogel	6	2.93	(56)
Rose Bengal	Sol–gel hybrid coatings based on alkyl silanes	4.4	4.4	(48)
Rose Bengal	P_mp_ (IRA900)	8	5.5	This work
Rose Bengal	P_gel_ (IRA400)	8	5.5	This work

**Figure 4:**
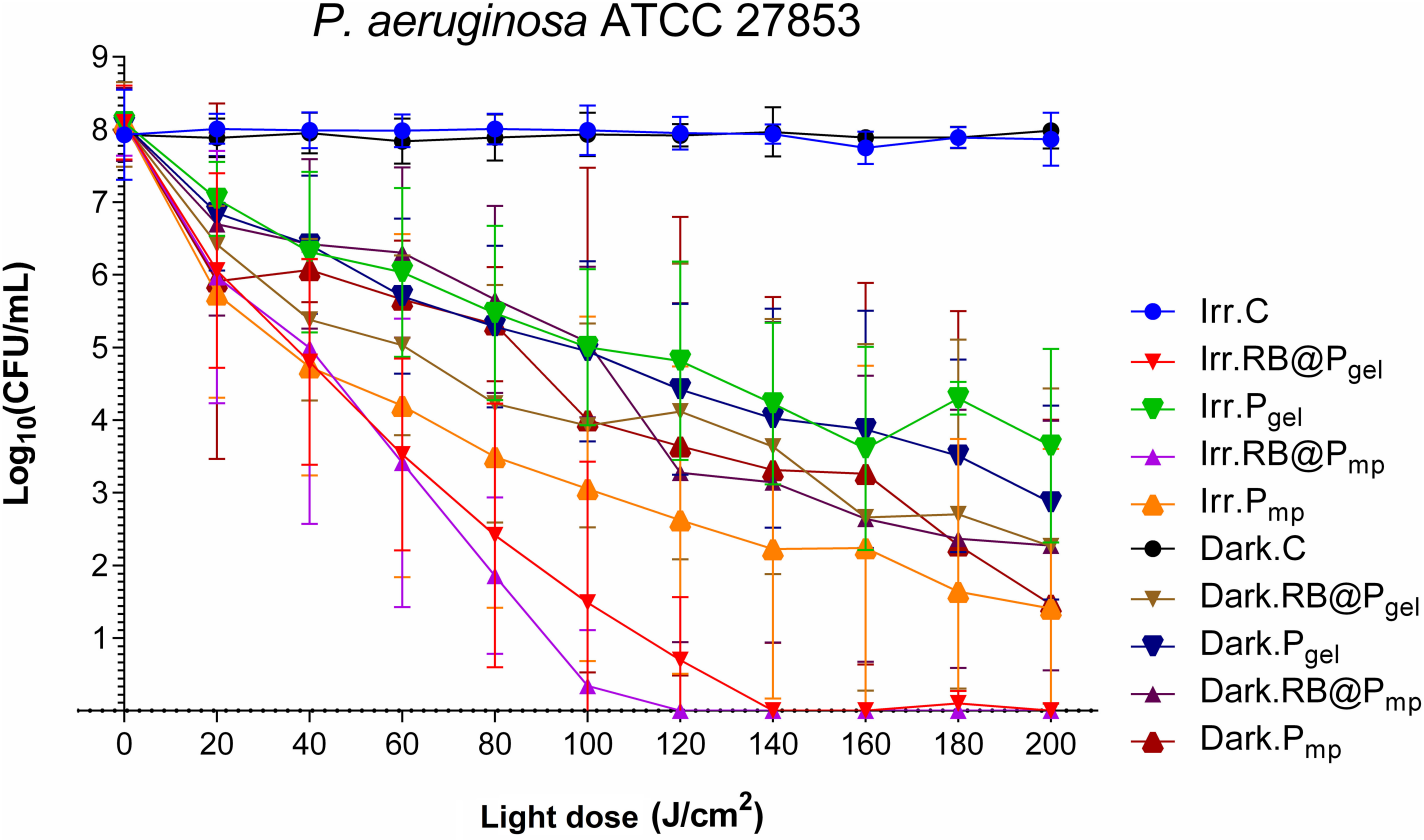
Survival curves corresponding to the photodynamic inactivation of *Pseudomonas aeruginosa*. Every *point* is the average of three independent experiments. *Error bars* correspond to the standard deviations. Legend titles: *Irr*, irradiated samples; *Dark*, controls in the darkness; *C*, control, only microbial suspension; *RB@P*_*gel*_, Amberlite® IRA-400 (P_gel_) loaded with Rose Bengal (RB); *P*_*gel*_, P_gel_ resin without RB; *RB@P*_*mp*_, Amberlite® IRA-900 (P_mp_) loaded with RB; *P*_*mp*_, P_mp_ resin without RB. Adapted from (34). Copyright 2020 with permission from Elsevier.

The activity against *P. aeruginosa* of RB@P_mp_ and RB@P_gel_ was recently reported by us (34) and is included in this study for comparison to the rest of the pathogens. A complete eradication of this species (8 log_10_ CFU/ml) was observed with both polymers when light was applied (Figure 4 and Supplementary Figures 2, 3). Also, an important dark toxicity of the polymers (~6 log_10_ CFU/ml reduction) was observed, indicating that the polymeric matrix is also playing an important role, probably due to the presence of the ammonium groups that can interact efficiently with the external wall of the bacterium cell (57). This activity is comparable to that reported for methylene blue encapsulated in porous silica nanoparticles (58) and for chitosan used as a carrier of Toluidine blue O (59) that also induced a reduction of 8 log_10_ CFU/ml, and for the aforementioned system involving indocyanine green loaded in superparamagnetic iron oxide nanoparticles, which induced a reduction of 12 log_10_ CFU/ml (53). Nevertheless, in these cases, the activity of the materials in the dark is negligible or very low. The corresponding comparative table for this bacterium can be found in the cited publication (34).

### Activity Against *Candida albicans*

**Figure 5:**
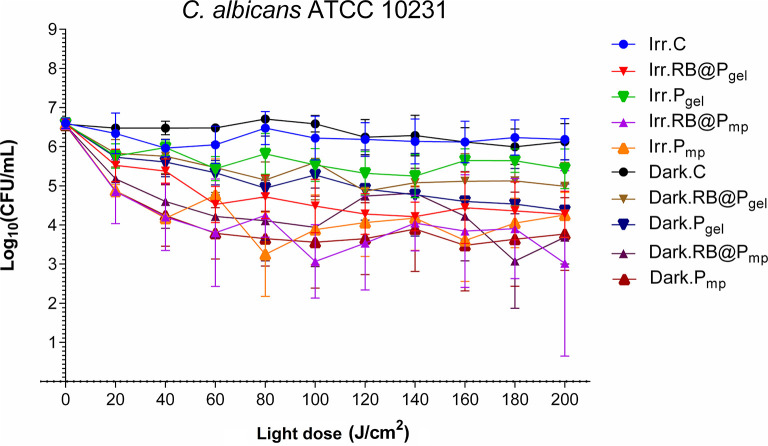
Survival curves corresponding to the photodynamic inactivation of *Candida albicans*. Every *point* is the average of three independent experiments. *Error bars* correspond to the standard deviations. Legend titles: *Irr*, irradiated samples; *Dark*, controls in the darkness; *C*, control, only microbial suspension; *RB@P*_*gel*_, Amberlite® IRA-400 (P_gel_) loaded with Rose Bengal (RB); *P*_*gel*_, P_gel_ resin without RB; *RB@P*_*mp*_, Amberlite® IRA-900 (P_mp_) loaded with RB; *P*_*mp*_, P_mp_ resin without RB.

The antifungal activity of polymers RB@P_mp_ and RB@P_gel_ was evaluated and the CFU per milliliter values after aPDI treatment presented in Figure 5 and Supplementary Figures 4, 5. Reductions of 1.5–3.0 log_10_ CFU/ml are observed against *C. albicans* for all the polymers, in both irradiated and dark conditions. It seems that some toxicity is related to the polymeric matrices P_mp_ and P_gel_; hence, RB direct photodynamic action seems to be not very important for *C. albicans*. As expected, light alone did not show any inhibition. The dark activity of the polymers (around 2.5 log_10_ CFU/ml) is probably connected to the presence of the positively charged groups on the surface of the polymer since several materials containing ammonium compounds have been reported to exhibit antifungal properties (60, 61). On the other hand, the scarce photoactivity of RB against *C. albicans* has been reported previously (62), which might probably rely on features such as the thickness of the yeast cell wall. However, it is not discarded for future studies that an increase in the concentration of the photosensitizer would lead to enhanced photoactivities. Finally, it can be said that a slightly better performance of the macroporous resin is observed in Figure 5 as compared to the gel-type one, probably due to the higher specific surface of the P_mp_ material.

Reports on the photoinactivation of *C. albicans* and other opportunistic *Candida* non-*albicans* species using photoactive solid materials are scarce. Table 4 summarizes some representative examples. The best results are obtained with a cationic phthalocyanine electrostatically attached to poly(propylene)-based films, which caused a 4 log_10_ decrease of the *C. albicans* population (65). Good results were observed as well when anionic porphyrin was used as a photosensitizer, but mainly when it was conjugated with platinum nanoparticles, showing a 3.95 log_10_ CFU/ml decrease (64).

**Table 4:** Representative examples reported in literature of *Candida albicans* inactivation caused by photosensitizing materials.

**Photosensitizer**	**Support**	**Initial load (log_**10**_ CFU/ml)**	**Load reduction (Δlog_**10**_ CFU/ml)**	**References**
Porphyrin	Polysilsesquioxane	6	2.5	(63)
Anionic porphyrin	Pt nanoparticles	8	3.95	(64)
Porphyrin	Silica-coated magnetite NPs	6	2.5	(55)
Cationic phthalocyanine	Poly(propylene)	6	4	(65)
Toluidine blue/Rose Bengal	Cellulose acetate	5.3	0.9	(66)
Rose Bengal	P_mp_ (IRA900)	6	3	This work
Rose Bengal	P_gel_ (IRA400)	6	1.5	This work

An important question that can arise, for all the microorganisms studied, is the potential formation of biofilms during the time that the experiment is running. Although this is always possible, (a) the continuous agitation of the samples minimizes this possibility and (b) typical conditions for biofilm formation like extended incubations (24–72 h) are avoided. Nevertheless, this fact should always be taken into account in studies involving surfaces.

Throughout this study, we are assuming that the killing of the microorganisms involves, very likely, singlet oxygen (type II mechanism), provided that RB is a well-known generator of this ROS upon visible light excitation in solution (67, 68). However, since some type I photoactivity has also been described for this photosensitizer (*via* superoxide anion) (69), this pathway cannot be ruled out completely in the complex environment created by the polymer matrix. Nevertheless, the existence of natural defensive agents like superoxide dismutase (SOD) makes the involvement of this ROS in the mechanism of cell death very unlikely. A more in-depth study would be needed to afford some clarification on this question, but this is out of the scope of this work.

## Conclusion

The aPDI capacity of RB@P_mp_ and RB@P_gel_ was addressed against both Gram-positive (*S. aureus* and *E. faecalis*) and Gram-negative (*E. coli* and *P. aeruginosa)* bacteria as well as the pathogenic yeast *C. albicans*. At a high total light dose (200 J/cm^2^), both groups of bacteria reduced their populations (5–8 log_10_ CFU/ml) in the presence of the photoactive polymers and light in a statistically significant manner (*p* < 0.01 to *p* < 0.0001, depending on the specific case; see Supplementary Material). Only for *C. albicans* was the observed photodynamic action scarce, although the effect of the polymeric matrix in the dark is the cause of around 2.5 log_10_ of CFU/ml reduction (statistically significant, with *p* < 0.05) and could be of interest for further studies.

Finally, we would like to stress that, only as a proof-of-concept, despite anionic photosensitizers, like RB, being largely considered ineffective for the inactivation of Gram-negative bacteria, we have shown that, when combined with commercial supports like cationic exchange resins, the resultant systems can be efficient materials against bacterial pathogens. The polymers described here lack the complexity of the other systems described in the literature, but it is precisely the accessibility of the starting materials that makes this combination an appealing option for new practical developments.

## Data Availability Statement

The original contributions presented in the study are included in the article/Supplementary Material, further inquiries can be directed to the corresponding author/s.

## Author Contributions

AR and FG designed and supervised the study. CAdV synthesized the materials under study. VP-L performed the biological experiments and conducted the statistical analysis of the data. RG, RdL, VP-L, JM, AR, and FG wrote parts of the manuscript. RG edited the manuscript. All authors read and approved the final version.

## Conflict of Interest

The authors declare that the research was conducted in the absence of any commercial or financial relationships that could be construed as a potential conflict of interest.
