# Supplementary material for: Broad-Spectrum Photo-Antimicrobial Polymers Based on Cationic Polystyrene and Rose Bengal
Source: Front Med (Lausanne). 2021 May 24;8:641646. doi: 10.3389/fmed.2021.641646 (PMC8180575; doi:10.3389/fmed.2021.641646)
Supplement: Supplementary file 1 [file Data_Sheet_1.PDF]

frontiers

Supplementary Material

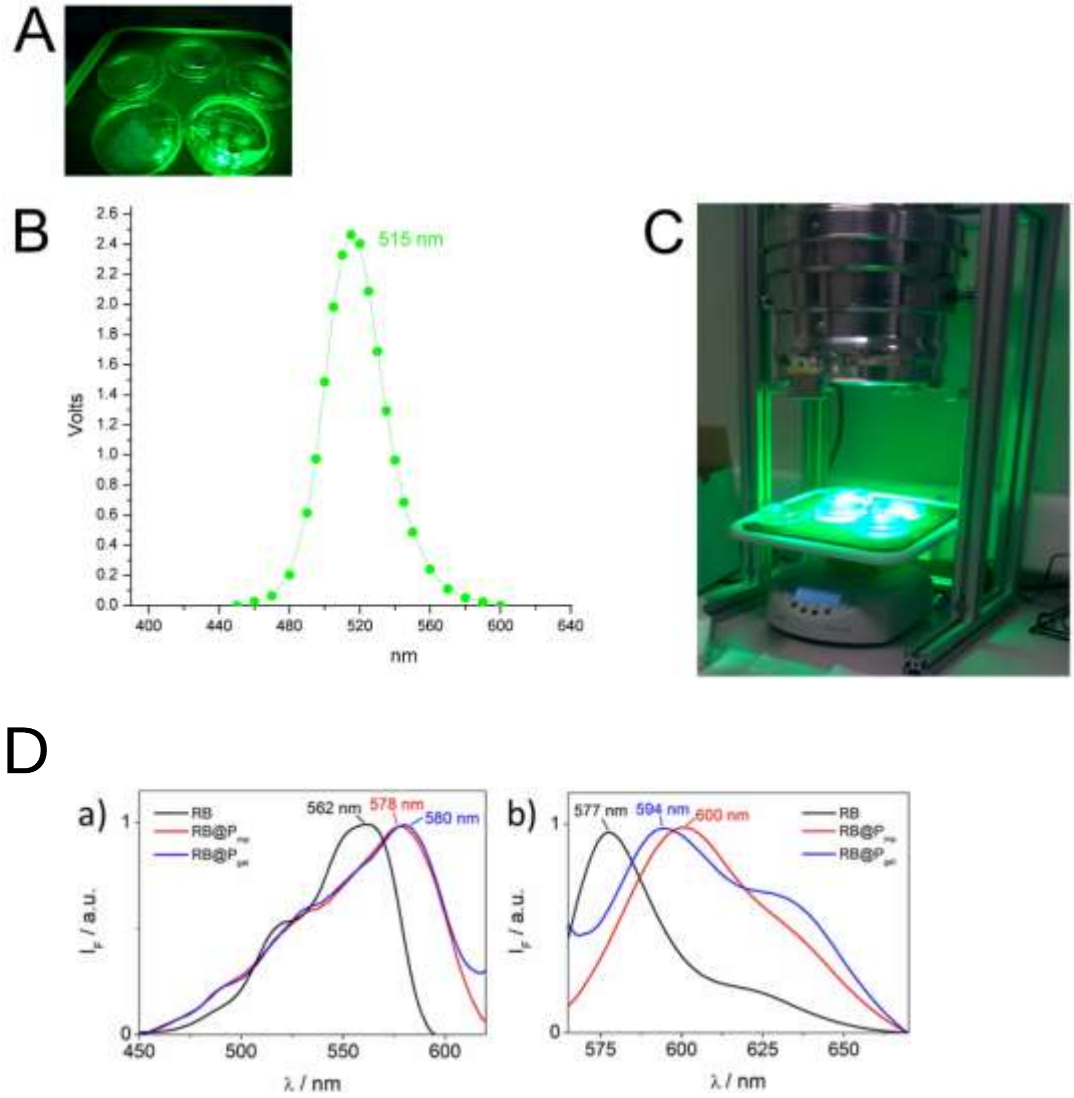

Supplementary Figure 1. A: Picture of 5 experimental groups subjected to irradiation. **B:** Green LED emission spectrum. **C:** Picture of the setup used. **D:** Spectroscopic characterization of the polymers used in this study (reprinted from ref. [1] with permission from Elsevier), (a) excitation spectra of **RB** in EtOH, **RB@P$_{mp}$** and **RB@P$_{gel}$** (both polymers in the solid state); Emission monitored at 600 nm. (b) Emission spectra of **RB** in EtOH, **RB@P$_{mp}$** and **RB@P$_{gel}$** (both polymers in the solid state); Excitation set at 550 nm.

[1] del Valle C.A., Pérez-Laguna V., Resta I.M., Gavara R., Felip-León C., Miravet J.F., Rezusta A., Galindo F. A cost-effective combination of Rose Bengal and off-the-shelf cationic polystyrene for the photodynamic inactivation of *Pseudomonas aeruginosa*. Mater. Sci. Eng. C (2020) 117:111302. doi:10.1016/j.msec.2020.111302

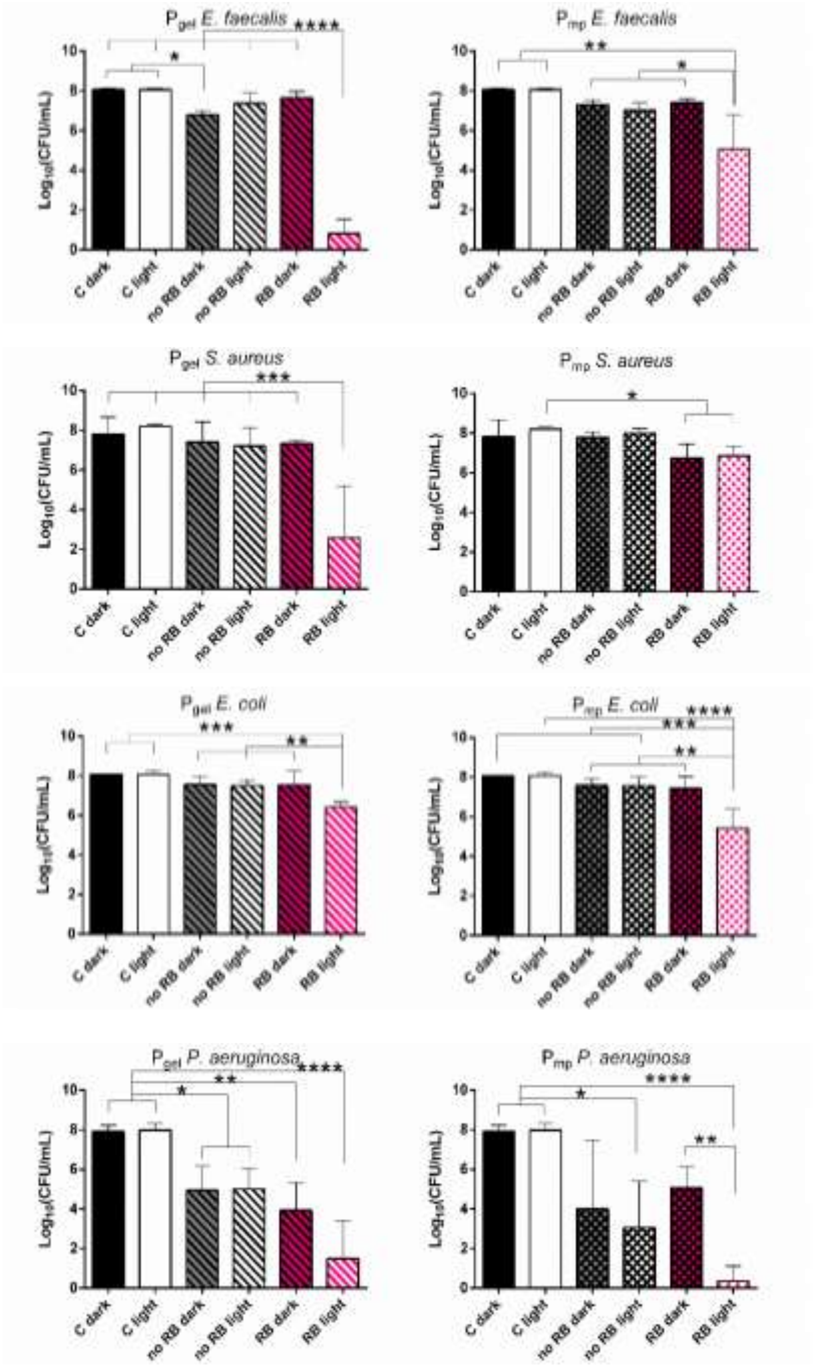

Supplementary Figure 2. Effects of the photodynamic activity of **RB@P$_{gel}$** (left) and **RB@P$_{mp}$** (right) on reduction of CFU/mL of Gram (+) bacteria, *E. faecalis* ATCC 29212 and *S. aureus* ATCC 29213, and Gram (-) bacteria, *E. coli* ATCC 25922 and *P. aeruginosa* ATCC 27853, at a total light dose of **100 J/cm^2**. In the graphs "C" refers to controls, *i.e*. microbial suspensions without any photosensitizer, "RB" refers to the polymers with Rose Bengal and "no RB" refers to **P$_{mp}$** or **P$_{gel}$** resin without Rose Bengal encapsulated. The error bars represent the standard deviation calculated for three measurements. * $p < 0.05$; **$p < 0.01$; ***$p < 0.001$; ****$p < 0.0001$. Data for *P. aeruginosa* reprinted from ref. [1] with permission from Elsevier.

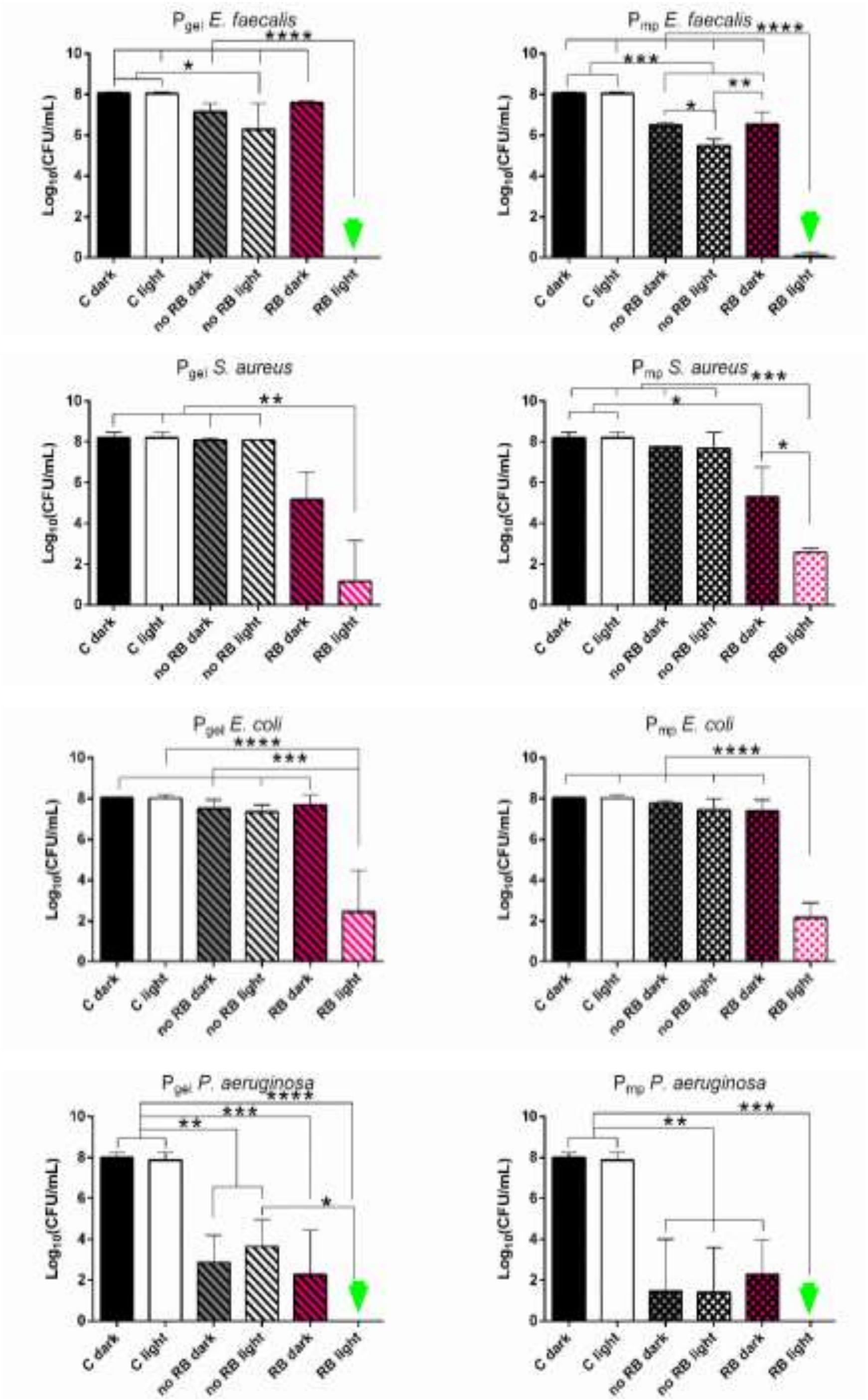

Supplementary Figure 3. Effects of the photodynamic activity of **RB@P_{gel}** (left) and **RB@P_{mp}** (right) on reduction of CFU/mL of Gram (+) bacteria, *E. faecalis* ATCC 29212 and *S. aureus* ATCC 29213, and Gram (-) bacteria, *E. coli* ATCC 25922 and *P. aeruginosa* ATCC 27853, at a total light dose of **$200\ J/cm^2$**. In the graphs "C" refers to controls, *i.e*. microbial suspensions without any photosensitizer, "RB" refers to the polymers with Rose Bengal and "no RB**"** refers to **P_{mp}** or **P_{gel}** resin without Rose Bengal encapsulated. The error bars represent the standard deviation calculated for three measurements. The green arrow indicates 99.999999% reduction of bacterial population. * $p < 0.05$; **$p < 0.01$; ***$p < 0.001$; ****$p < 0.0001$. Data for *P. aeruginosa* reprinted from ref. [1] with permission from Elsevier.

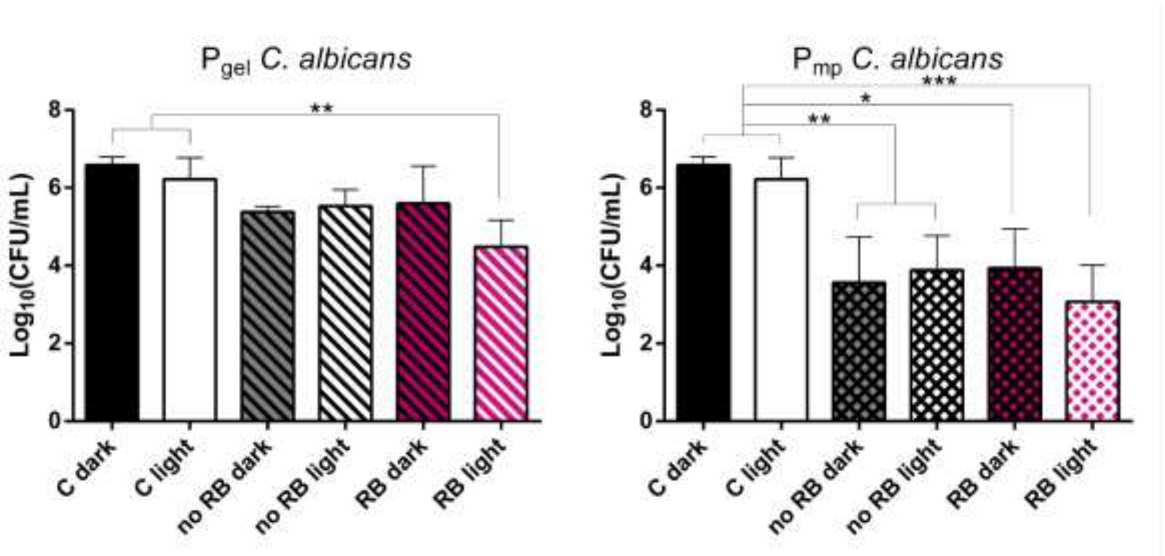

Supplementary Figure 4. Effects of the photodynamic activity of **RB@P$_{gel}$** (left) and **RB@P$_{mp}$** (right) on reduction of CFU/mL of *Candida albicans* ATCC 10231 at a total light dose of **100 J/cm^2**. In the graphs, "C" refers to controls, *i.e*. microbial suspensions without any photosensitizer and "RB" refers to the polymers with Rose Bengal and "no RB" refers to **P$_{mp}$** or **P$_{gel}$** resin without Rose Bengal encapsulated. The error bars represent the standard deviation calculated for three measurements. $*p < 0.05$; $**p < 0.01$; $***p < 0.001$.

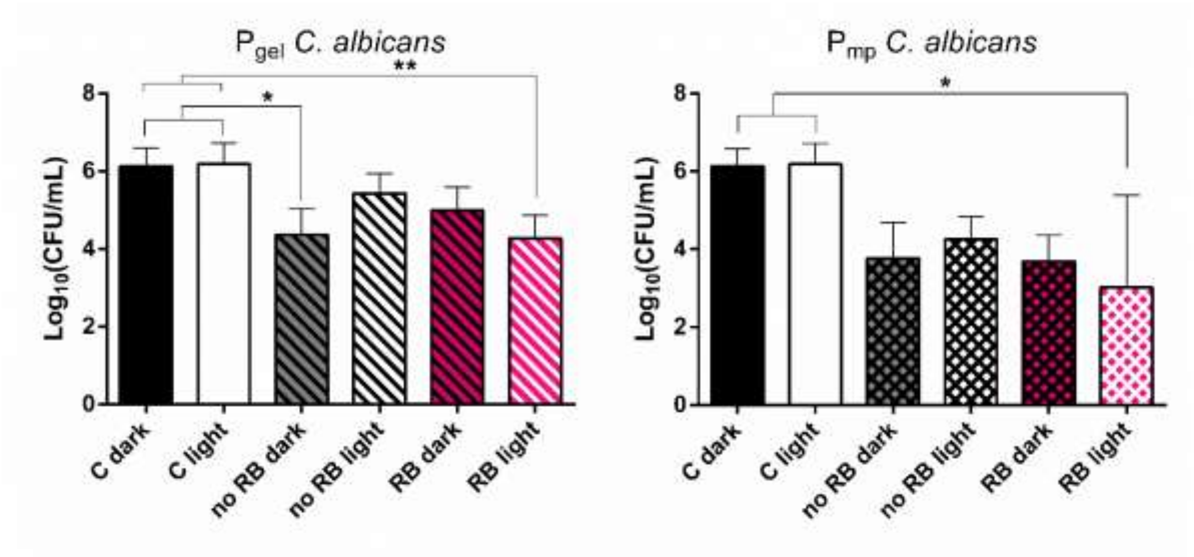

Supplementary Figure 5. Effects of the photodynamic activity of **RB@P$_{gel}$** (left) and **RB@P$_{mp}$** (right) on reduction of CFU/mL of *Candida albicans* ATCC 10231 at a total light dose of **200 J/cm^2**. In the graphs, "C" refers to controls, *i.e*. microbial suspensions without any photosensitizer and "RB" refers to the polymers with Rose Bengal and "no RB" refers to **P$_{mp}$** or **P$_{gel}$** resin without Rose Bengal encapsulated. The error bars represent the standard deviation calculated for three measurements. $*p < 0.05$; $**p < 0.01$.
